# Latent class analysis for identification of sub-phenotypes predicting prognosis in hospitalized out-of-hospital cardiac arrest

**DOI:** 10.2478/jccm-2025-0016

**Published:** 2025-04-30

**Authors:** Yuki Kishihara, Hideto Yasuda, Masahiro Kashiura, Shunsuke Amagasa, Hiroyuki Tamura, Yutaro Shinzato, Takashi Moriya

**Affiliations:** Department of Emergency and Critical Care Medicine, Jichi Medical University Saitama Medical Center, Saitama, Japan; Department of Clinical Research Education and Training Unit, Keio University Hospital Clinical and Translational Research Center, Tokyo, Japan; School of Nursing, Midwifery and Social Work, UQ Centre for Clinical Research, The University of Queensland, Australia; School of Nursing and Midwifery; Alliance for Vascular Access Teaching and Research, Griffith University, Australia; Department of Emergency and Transport Medicine, National Center for Child Health and Development, Tokyo, Japan

**Keywords:** adult, cardiopulmonary resuscitation, latent class analysis, out-of-hospital cardiac arrest, prognosis

## Abstract

**Aim of the study:**

To determine which out-of-hospital cardiac arrest (OHCA) patients should receive advanced treatment is extremely challenging. The objective was to identify sub-phenotypes predicting the prognoses of adult OHCA patients by latent class analysis (LCA) using data up to just after admission.

**Material and Methods:**

We conducted a retrospective observational study using multicentre OHCA registry from 95 Japanese hospitals including adult non-traumatic hospitalized OHCA. The primary outcome was 30-day favourable neurological outcome. Our LCA used clinically relevant variables up to just after admission and the optimal class number was determined from clinical importance and Bayesian information criterion. The associations between subphenotypes and outcomes were analysed using univariate logistic regression analysis with odds ratios (ORs) and 95% confidence intervals (CIs).

**Results:**

Our LCA included 2,162 patients and identified four sub-phenotypes. The base excess on hospital arrival had the highest discriminative power. Thirty-day favourable neurological outcomes were observed in 526 patients (24.3%), including 284 (53.8%) in Group 1, 179 (21.2%) in Group 2, 26 (11.4%) in Group 3, and 37 (6.6%) in Group 4. Prehospital return of spontaneous circulation (ROSC) was achieved in 1,009 patients (46.7%), including 379 (81.8%) in Group 1, 340 (40.3%) in Group 2, 115 (50.4%) in Group 3, and 175 (31.1%) in Group 4. Univariate logistic regression analysis for primary outcome using Group 4 as reference revealed ORs (95% CI) of 16.5 (11.4–24.1) in Group 1, 3.83 (2.64–5.56) in Group 2, and 1.83 (1.08–3.10) in Group 3.

**Conclusions:**

Our LCA classified OHCA into four sub-phenotypes showing significant differences for prognosis. In cases who achieved prehospital ROSC, it might be meaningful to continue advanced therapeutic interventions.

## Introduction

Out-of-hospital cardiac arrest (OHCA) remains responsible for numerous deaths [1]. Patients with OHCA are heterogeneous, with diverse factors that may be associated with prognosis [2,3]. Therefore, even if patients survive and are hospitalized (i.e., do not die in the emergency room), it is challenging to identify patients who should continue advanced therapeutic interventions that are resource-intensive, associated with a high economic burden, and not indiscriminately applicable to all patients [4,5].

Latent class analysis (LCA) may be useful for identifying sub-phenotypes to predict the prognoses of OHCA patients. A previous LCA for OHCA patients with initial shockable rhythms revealed three sub-phenotypes that were associated with prognosis [6]. Similarly, performing LCA for hospitalized OHCA patients may reveal certain sub-phenotypes that are associated with prognosis. This sub-phenotyping may prove helpful when clinicians consider which patients should receive more aggressive treatment.

If the present study identifies sub-phenotypes associated with prognoses, its findings may help better inform clinical decision-making for advanced therapeutic intervention administration that may help to achieve significant healthcare cost reductions. Therefore, our objective was to perform LCA to identify sub-phenotypes and evaluate the correlation between the sub-phenotypes and prognosis in hospitalized OHCA patients.

## Materials and methods

### Study Design

A multicentre retrospective observational study was conducted using the OHCA registry administered by the Japanese Association for Acute Medicine (JAAM). This registry collected pre- and post-hospital information on OHCA patients transported to 95 hospitals in Japan between 1 June 2014 and 31 December 2020. Pre- and post-hospital information was collected from the All-Japan Utstein Registry of the Fire and Disaster Management Agency, and medical personnel at each institution, respectively. This information was registered in a web-based system by the medical personnel at each institution, and the outcome assessors were not blinded.

The ethics committee of Jichi Medical University Saitama Medical Centre approved this specific study (approval number: S22-002). The informed consent was waived because the study involved no interventions diverged from standard cardiopulmonary resuscitation (CPR) practices. However, we provided an opt-out procedure on the website of the Department of Emergency Medicine of Jichi Medical University Saitama Medical Centre. This study was conducted according to the guidelines of the strengthening the reporting of observational studies in epidemiology statement and REporting of studies Conducted using Observational Routinely-collected health Data Statement, as well as the principles of the Declaration of Helsinki (Additional file 1) [7].

### Participants

We included OHCA patients performed CPR by emergency medical services (EMS) personnel. The exclusion criteria were as follows: 1) traumatic OHCA, 2) age < 18 years, 3) dead in ER, 4) inconsistent time from awareness to initiation of CPR or time from EMS contact to hospital arrival (i.e. negative values or outlier values > 2× the third quartile range—based on the findings of a previous study), and 5) missing data concerning LCA variables or outcomes [8].

### Data Collection

The following data were collected: age, sex, time of emergency call (7:00–14:59, 15:00–22:59, or 23:00–6:59 h), witness status (none, EMS personnel, others), bystander CPR (presence, absence, presence including rescue breathing), initial monitored cardiac rhythm (ventricular fibrillation [VF], pulseless ventricular tachycardia [VT], pulseless electrical activity [PEA], asystole, or other), cause of cardiac arrest (cardiogenic, respiratory, other intrinsic factors), time from call to initiation of CPR, pre-hospital adrenaline administration, pre-hospital shock delivery, pre-hospital advanced airway management (AAM; laryngeal mask, oesophageal obturator, or endotracheal tube), transportation by vehicular or air ambulance with a physician, time from EMS contact to hospital arrival, pre-hospital return of spontaneous circulation (ROSC), Glasgow coma scale (GCS) score on hospital arrival, blood gas findings on hospital arrival (pH, PaCO_2_, HCO_3_^−^, base excess [BE], and lactic acid [Lac]), extracorporeal membrane oxygenation (ECMO), intra-aortic balloon pumping (IABP), percutaneous coronary intervention (PCI), targeted temperature management (TTM), blood gas findings on admission (pH, PaO_2_, PaCO_2_, HCO_3_^−^, BE, Lac), 30-day cerebral performance category (CPC), and survival [9]. Based on a previous study, the times of emergency calls were categorised as 7:00–14:59, 15:00–22:59, and 23:00–6:59 h [10]. AAM included supraglottic airway and tracheal intubation, as both are considered to have comparable efficacy in terms of prognosis [11].

### Outcome Measures

The primary outcome was 30-day favourable neurological outcomes after cardiac arrest. A favourable neurological outcome was defined as a CPC score of 1 or 2 [9]. The secondary outcome was 30-day survival.

### Statistical Analysis

The following variables up to just after admission were selected for the LCA: age, sex, time of emergency call, tertiles of prefecture performance rates of out-of-hospital AAM, witness status, bystander CPR, initial cardiac rhythm, cause of OHCA, time from call to CPR, pre-hospital treatment (adrenaline, shock delivery, AAM), transportation by vehicular or air ambulance, time from EMS contact to hospital arrival, pre-hospital ROSC, GCS on hospital arrival, blood gas findings on hospital arrival or admission, ECMO, IABP, PCI, and TTM. In Japan, a few regional differences exist in terms of CPR practices. To account for these differences, we divided the prefectures into tertiles based on the AAM rate, according to a previously described approach [12]. The JAAM-OHCA registry involves a diverse array of institutions across Japan, and data collection was conducted according to each institution’s protocols. The data missingness unlikely occurred due to specific reasons related to outcomes, but were considered to be missing at random. Therefore, we used only complete cases for the statistical analyses.

Cluster analysis was conducted using 2–5 classes to explore the range of potential sub-phenotypes, however, there is no established method for calculating the sample size for LCA. The optimal number of clinically meaningful sub-phenotypes was determined by considering clinical importance and Bayesian Information Criterion (BIC), and the discriminative power of each variable was estimated using the maximum integrated complete-data likelihood criterion. A higher variable index for this parameter indicates a stronger association between the variable and the clustering process.

Kruskal-Wallis rank-sum or Chi-squared tests was performed to compare the differences in each variable between the sub-phenotypes, where continuous variables were described as medians and interquartile ranges (IQRs), and categorical variables as absolute counts and percentages (%). Univariate logistic regression analysis was performed to evaluate the association between the sub-phenotypes and prognosis, and crude odds ratios (ORs) with 95% confidence intervals (CIs) were calculated. Adjusting for other covariates was refrained, because sub-phenotypes are considered to represent underlying homogeneous groups within patient characteristics, which are not directly observable through the covariates^7^. And multivariate analysis using ECMO, IABP, and TTM as covariates was not performed, since our intention was to conduct an LCA to predict prognosis using data up to just after admission. Consequently, these covariates do not act as confounders in the association between sub-phenotypes and outcomes.

We used R statistical software version 4.1.3 (The R Project for Statistical Computing, Vienna, Austria) with the VarSelLCM package for statistical analyses, and two-sided p-values of < 0.05 were considered statistically significant.

## Results

### Patient Enrolment

**Fig. 1.**
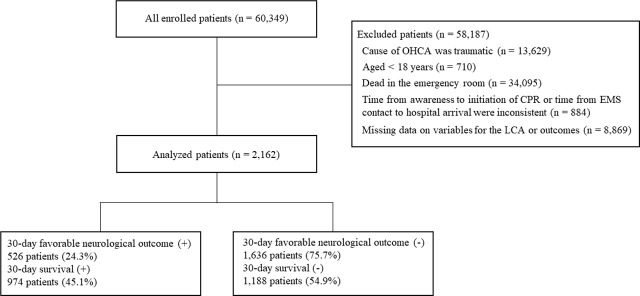
**Flowchart detailing the screening and enrolment process for this study.** Abbreviations: CPR, cardiopulmonary resuscitation; EMS, emergency medical service; LCA, latent class analysis; OHCA, out-of-hospital cardiac arrest.

A total of 60,349 patients were included and 2,162 patients were analysed (Figure 1). Details regarding reasons for exclusion are described in Figure 1.

### LCA

Four sub-phenotypes were identified (Supplementary Table 1 in Additional file 2). The patient characteristics in each sub-phenotypes are shown in Table 1, and the missing values are shown in Supplementary Table 2 in Additional file 2.

**Table 1.** Demographics and characteristics of analyzed patients with stratified by sub-phenotypes

**Variables**	**Overall (n=2,162)**	**Sub-phenotypes**				**p value**
		**Group 1 (n=528)**	**Group 2 (n=843)**	**Group 3 (n=228)**	**Group 4 (n=563)**	
Age, years, median (IQR)	68 (56–77)	68 (57–77)	64 (52–74)	75 (64–83)	69 (58–80)	< .01

Male, n (%)	1,553 (71.8)	389 (73.7)	649 (77.0)	138 (70.5)	377 (67.0)	< .01

Time of emergency call, n (%)						0.19
7:00–14:59	999 (46.2)	252 (47.7)	385 (45.7)	97 (42.5)	265 (47.1)	
15:00–22:59	758 (35.1)	195 (36.9)	290 (34.4)	77 (33.8)	196 (34.8)	
23:00–6:59	405 (18.7)	81 (15.3)	168 (19.9)	54 (23.7)	102 (18.1)	

Tertiles of prefecture performance rates of out-of-hospital advanced airway management, n (%)						
< 37.0	521 (24.1)	114 (21.6)	204 (24.2)	63 (27.6)	140 (24.9)	
37.0–63.6	981 (45.4)	253 (47.9)	386 (45.8)	92 (40.4)	250 (44.4)	0.53
> 63.6	660 (30.5)	161 (30.5)	253 (30.0)	73 (32.0)	173 (30.7)	

Witness status, n (%)						< .01
None	569 (26.3)	113 (21.4)	204 (24.2)	70 (30.7)	182 (32.3)	
Others	1327 (61.4)	369 (69.9)	552 (65.5)	121 (53.1)	285 (50.6)	
EMS personnel	266 (12.3)	46 (8.7)	87 (10.3)	37 (16.2)	96 (17.1)	

Bystander CPR, n (%)						< .01
Absence	1109 (51.3)	237 (44.9)	401 (47.6)	142 (62.3)	329 (58.4)	
Presence	795 (36.8)	227 (43.0)	331 (39.3)	68 (29.8)	169 (30.0)	
Presence including rescue breathing	258 (11.9)	64 (12.1)	111 (13.2)	18 (7.9)	65 (11.5)	

Initial monitored cardiac rhythm, n (%)						< .01
VF	868 (40.1)	332 (62.9)	409 (48.5)	34 (14.9)	93 (16.5)	
Pulseless VT	12 (0.6)	3 (0.6)	5 (0.6)	1 (0.4)	3 (0.5)	
PEA	607 (28.1)	105 (19.9)	231 (27.4)	82 (36.0)	189 (33.6)	
Asystole	517 (23.9)	37 (7.0)	157 (18.6)	87 (38.2)	236 (41.9)	
Other	158 (7.3)	51 (9.7)	41 (4.9)	24 (10.5)	42 (7.5)	

Cause of cardiac arrest, n (%)						< .01
Cardiogenic	1541 (71.3)	449 (85.0)	649 (77.0)	121 (53.1)	322 (57.2)	
Respiratory	188 (8.7)	16 (3.0)	46 (5.5)	50 (21.9)	76 (13.5)	
Other intrinsic disease	433 (20.0)	63 (11.9)	148 (17.6)	57 (25.0)	165 (29.3)	

Time from call to I-CPR, min, median (IQR)	9 (7–11)	8 (6–10)	8 (7–11)	9 (8–11.3)	9 (8–12)	< .01

Prehospital adrenaline administration, n (%)	767 (35.5)	111 (21.0)	282 (33.5)	120 (52.6)	254 (45.1)	< .01

Prehospital shock delivery, n (%)	1027 (47.5)	371 (70.3)	478 (56.7)	48 (21.1)	130 (23.1)	< .01

Prehospital AAM, n (%)	1251 (57.9)	220 (41.7)	486 (57.7)	161 (70.6)	384 (68.2)	< .01

Physician during emergency transport, n (%)	1860 (86.0)	437 (82.8)	715 (84.8)	201 (88.2)	507 (90.1)	0.01

Time from EMS contact to HA, min, median (IQR)	23 (18–29)	23 (17–28)	21 (16–27)	27 (21–35)	25 (19–30)	< .01

Prehospital ROSC, n (%)	1,009 (46.7)	379 (81.8)	340 (40.3)	115 (50.4)	175 (31.1)	< .01

GCS on HA, median (IQR)	3 (3–3)	3 (3–6)	3 (3–3)	3 (3–3)	3 (3–3)	< .01

pH on HA, median (IQR)	7.07 (6.90–7.24)	7.30 (7.25–7.36)	7.06 (6.95–7.17)	7.04 (6.93–7.19)	6.83 (6.73–6.93)	< .01

PaCO_2_ on HA, mmHg, median (IQR)	53.3 (38.8–76.9)	39.1 (33.8–46.3)	55.6 (40.2–74.0)	76.9 (59.2–101.6)	69.1 (49.6–93.1)	< .01

HCO_3_^−^ on HA, mmol/l, median (IQR)	15.6 (12.0–19.2)	19.3 (17.0–21.6)	14.8 (12.6–17.4)	21.6 (16.7–25.7)	10.8 (8.0–13.8)	< .01

BE on HA, median (IQR)	−14.2 (−20.2 –−8.4)	−6.3 (−9.0 –−3.8)	−15.0 (−17.7 –−11.9)	−8.4 (−14.7 –−2.1)	−22.7 (−26.0 –−19.7)	< .01

Lac on HA, mg/dl, median (IQR)	93.6 (63.0–127.6)	57.0 (38.0–73.0)	96.0 (73.8–117.9)	100.9 (70.4–144.0)	133.0 (108.9–162.0)	< .01

ECMO, n (%)	1656 (76.6)	445 (84.3)	567 (67.3)	202 (88.6)	442 (78.5)	< .01

IABP, n (%)	1601 (74.1)	391 (74.1)	561 (66.5)	201 (88.2)	448 (79.6)	< .01

PCI, n (%)	1610 (74.5)	343 (65.0)	587 (69.6)	205 (89.9)	475 (84.4)	< .01

TTM, n (%)	1246 (57.6)	239 (45.3)	427 (50.7)	166 (72.8)	414 (73.5)	< .01

pH on admission, median (IQR)	7.30 (7.19–7.37)	7.37 (7.33–7.42)	7.30 (7.24–7.37)	7.33 (7.21–7.41)	7.11 (6.99–7.20)	< .01

PaO_2_ on admission, mmHg, median (IQR)	148.0 (95.0–252.0)	145.1 (101.0–230.4)	150.0 (93.6–255.5)	124.2 (82.8–198.0)	155.0 (95.8–272.5)	0.01

PaCO_2_ on admission, mmHg, median (IQR)	41.0 (34.4–49.2)	39.9 (35.3–43.9)	39.6 (33.1–46.9)	48.5 (38.7–62.0)	44.4 (34.1–59.0)	< .01

HCO_3_^−^ on admission, mmol/l, median (IQR)	19.6 (15.6–22.9)	22.4 (20.6–24.2)	19.3 (16.6–22.1)	26.0 (20.6–29.4)	13.9 (10.8–16.6)	< .01

BE on admission, median (IQR)	−6.0 (−11.4 –−2.1)	−2.3 (−4.1 –−0.5)	−6.5 (−9.4 –−3.5)	−0.3 (−5.3 – 4.0)	−15.0 (−18.6 –−11.1)	< .01

Lac on admission, mg/dl, median (IQR)	48.0 (21.6–84.6)	21.0 (13.5–32.6)	45.0 (26.0–68.0)	52.6 (21.5–115.2)	95.2 (64.8–130.5)	< .01

AAM, advanced airway management; BE, base excess; CPR, cardiopulmonary resuscitation; ECMO, extracorporeal membrane oxygenation; EMS, emergency medicine service; GCS, glasgow coma scale; HA, Hospital arrival; IABP, intra-aortic balloon pumping; I-CPR, Initiation of CPR; IQR, interquartile range; Lac, lactic acid; min, minutes; PCI, percutaneous coronary intervention; PEA, pulseless electrical activity; ROSC, return of spontaneous circulation; TTM, targeted temperature management; VF, ventricular fibrillation; VT, ventricular tachycardia.

**Fig. 2.**
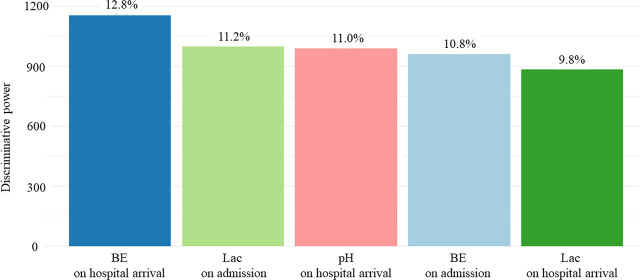
**The five factors with high discriminative power in the LCA.** Abbreviations: BE, base excess; Lac, lactic acid; LCA, latent class analysis. All of these variables were blood gas findings.

The factor with the highest discriminative power was BE on hospital arrival (Figure 2). The distribution of values was as follows: Group 1, median (IQR) of BE on hospital arrival was −6.3 (−9.0 to −3.8); Group 2, −15.0 (−17.7 to −11.9); Group 3, −8.4 (−14.7 to −2.1); and Group 4, −22.7 (−26.0 to −19.7) (Table 1, Supplementary Figure 1a,b in Additional file 2). Moreover, 332 patients (62.9%) had VF as initial cardiac rhythm in Group 1, 409 (48.5%) in Group 2, 34 (14.9%) in Group 3, and 93 (16.5%) in Group 4; 449 (85.0%) had cardiogenic cardiac arrest in Group 1, 649 (77.0%) in Group 2, 121 (53.1%) in Group 3, and 322 (57.2%) in Group 4; 379 (81.8%) experienced prehospital ROSC in Group 1, 340 (40.3%) in Group 2, 115 (50.4%) in Group 3, and 175 (31.1%) in Group 4 (Table 1, Supplementary Figure 1c–e in Additional file 2).

### Sub-phenotypes and Outcomes

Favourable 30-day neurological outcomes were observed in 526 patients (24.3%): 284 (53.8%) in Group 1, 179 (21.2%) in Group 2, 26 (11.4%) in Group 3, and 37 (6.6%) in Group 4 (p < .01; Table 2). Thirty-day survival was observed in 974 patients (45.1%): 395 (74.8%) in Group 1, 407 (48.3%) in Group 2, 76 (33.3%) in Group 3, and 96 (17.1%) in Group 4 (p < 0.01; Table 2).

**Table 2.** Outcomes distribution in each sub-phenotype

	**Overall (n=2,162)**	**Sub-phenotypes**				**p value**
		**Group 1 (n=528)**	**Group 2 (n=843)**	**Group 3 (n=228)**	**Group 4 (n=563)**	
30-day favorable neurological outcome, n (%)	526 (24.3)	284 (53.8)	179 (21.2)	26 (11.4)	37 (6.6)	< .01
30-day survival, n (%)	974 (45.1)	395 (74.8)	407 (48.3)	76 (33.3)	96 (17.1)	< .01

Univariate logistic regression analysis for 30-day favourable neurological outcomes using Group 4 as the reference revealed ORs (95% CIs) of 16.5 (11.4–24.1) in Group 1, 3.83 (2.64–5.56) in Group 2, and 1.83 (1.08–3.10) in Group 3 (Table 3). For 30-day survival, the ORs and 95% CIs were 14.4 (10.8–19.4) in Group 1, 4.54 (3.51–5.88) in Group 2, and 2.43 (1.71–3.46) in Group 3 (Table 3).

**Table 3.** Logistic regression analysis for the correlation between sub-phenotypes and outcomes

**Sub-phenotypes**	**Crude OR**	**95% CI (lower)**	**95% CI (upper)**	**p value**
30-day favorable neurological outcome				
Group 1	16.5	11.4	24.1	< .01
Group 2	3.83	2.64	5.56	< .01
Group 3	1.83	1.08	3.10	0.02
Group 4	ref	ref	ref	-

30-day survival				
Group 1	14.4	10.8	19.4	< .01
Group 2	4.54	3.51	5.88	< .01
Group 3	2.43	1.71	3.46	< .01
Group 4	ref	ref	ref	-

Abbreviations: CI, confidence interval; OR, odds ratio.

## Discussion

The current LCA was performed on adult OHCA hospitalized patients and four sub-phenotypes were identified with prognosis being distributed among them with statistically significant differences. Univariate logistic regression analysis using Group 4 as the reference showed that Groups 1, 2, and 3 were significantly associated with prognosis. The factor with the highest discriminative power was BE on hospital arrival.

There are several possible interpretations for the results of the current study. The statistically significant differences in the incidence rates of prognosis between the sub-phenotypes may be attributed to whether ROSC was achieved before hospital arrival. In the current study, cardiogenic cardiac arrest, an initial shockable rhythm, and pre-hospital ROSC were more common in Group 1. A previous study reported that cardiogenic cardiac arrest is a common cause of shockable rhythm, and that OHCA patients with shockable rhythms are more likely to achieve ROSC before hospital arrival [13]. Studies have also reported that OHCA patients who achieve ROSC before hospital arrival have better prognoses [13,14]. Therefore, in the current study, cases expected to achieve pre-hospital ROSC, such as cardiogenic cardiac arrest or initial shockable rhythms, were classified into Group 1, and Group 1 may have had the highest number of patients with a favourable prognosis. And there was a significant difference in the haemodynamics between those patients who achieved ROSC before hospital arrival and those who did not. As a result, blood gas findings may have had high discriminative power in the current study.

On the other hand, the relatively high incidence of outcomes of the current study may have facilitated the distinctive sub-phenotyping into four groups. A previous LCA of OHCA with initial shockable rhythms with the overall 30-day survival rate of ~30% identified three sub-phenotypes, where 30-day survival rates were 85.9%, 30.7%, and 15.7% [6]. The current study with the overall 30-day survival rate of 45.1% identified four sub-phenotypes, where 30-day survival rates were 74.8%, 48.3%, 33.3%, and 17.1%. The current LCA shows a similar trend to the previous LCA, and the distribution of factors such as age, witness status, presence of bystander CPR, and initial monitored cardiac rhythm were similar^6^. Conversely, another previous LCA of OHCA with non-shockable rhythms with the overall 30-day favourable neurological outcome rate of ~2% identified four sub-phenotypes, where 30-day favourable neurological outcome rates were 6.6%, 1.2%, 0.7%, and 0.1%, whereas 30-day favourable neurological outcomes of the current LCA was 24.3% [15]. These findings suggest that LCA reveals larger differences in outcome incidence rates between sub-phenotypes when the overall incidence rate is high, and smaller differences when the overall incidence rate is low [16].

The current study is novel in using an uncommon approach for predicting the prognoses of adult OHCA hospitalized patients. The LCA classified patients into four sub-phenotypes that showed statistically significant differences in prognosis. The results of current LCA suggest that patients who achieved prehospital ROSC, specifically those suspected of cardiogenic cardiac arrest or those with an initial shockable rhythm, may have a better prognosis. In such cases, it is meaningful to continue advanced therapeutic interventions.

The current LCA has several limitations. First, the results may lack external validity. Owing to technical limitations, external validity verification using the bootstrap method was not performed. Therefore, the results may not be generalisable to other patient populations. Furthermore, the findings of current study cannot be applied to regions where interventions for patients with OHCA are legally mandated. For example, in Romania, all individuals who experience OHCA must receive advanced resuscitation measures and life-sustaining interventions without discrimination based on prognosis or resource allocation. Therefore, the results of current study cannot be applied to regions such as Romania, and there may be concerns regarding the external validity of current study. Second, owing to the insufficient sample size, the class separation achieved by the current LCA may have been low. Although no standardised criteria are available for determining optimal LCA sample sizes, statistical discussions have suggested that increasing the sample size may improve the class separation achieved by LCA [16]. Therefore, increasing the sample size and conducting LCA may yield different results. Finally, the results of this study may have been incorrect, owing to an insufficient number of factors incorporated into the LCA. Previous studies have reported that patients with low activities of daily living (ADL) before OHCA may have poorer prognoses [17]. However, this factor was not accounted for in the JAAM-OHCA registry, and was not included in the current LCA. Therefore, future LCAs conducted using other factors that correlate with prognosis such as ADL before OHCA may allow for more appropriate sub-phenotyping.

## Conclusions

The current LCA in adult OHCA hospitalized patients identified four sub-phenotypes based on statistically significant differences related to prognosis. The subphenotypes may help to inform future clinical decision-making, such as in cases who achieved prehospital ROSC, it might be meaningful to continue advanced therapeutic interventions and have the potential to reduce healthcare costs.
